# Chronic Stable Angina Is Associated with Lower Health-Related Quality of Life: Evidence from Chinese Patients

**DOI:** 10.1371/journal.pone.0097294

**Published:** 2014-05-19

**Authors:** Jing Wu, Yuerong Han, Judy Xu, Yang Lu, Hongliang Cong, Junyi Zheng, He Sun

**Affiliations:** 1 School of Pharmaceutical Science and Technology, Tianjin University, Tianjin, China; 2 School of Public Administration, Southwest University of Finance and Economics, Chengdu, China; 3 Harbor UCLA Medical Center, Torrance, California, United States of America; 4 Tianjin Chest Hospital, Tianjin, China; Kurume University School of Medicine, Japan

## Abstract

**Objectives:**

To compare health-related quality of life (HRQoL) between patients with stable angina and the general population in China and to examine factors associated with HRQoL among patients with stable angina.

**Methods:**

A cross-sectional HRQoL survey of stable angina patients recruited from 4 hospitals (n = 411) and the general population recruited from 3 Physical Examination Centers (n = 549) was conducted from July to December, 2011 in two large cities, Tianjin and Chengdu. HRQoL was assessed using the EQ-5D, EQ-VAS, and SF-6D instruments. The health status specific to patients with stable angina was assessed using the Seattle Angina Questionnaire (SAQ). Information on socio-demographic, clinical, and lifestyle factors were also collected. Nested regressions were performed to explore how these factors were associated with HRQoL in patients with stable angina.

**Results:**

Compared with the general population (44.2±10 years, 49.9% females), stable angina patients (68.1±12 years, 50.4% females) had significantly lower HRQoL scores in EQ-5D utility index (0.75±0.19 vs. 0.90±0.20, p<0.05), SF-6D utility index (0.68±0.12 vs. 0.85±0.11, p<0.05), and EQ-VAS (71.2±12.3 vs. 83.9±10.9, p<0.05). The differences remained (−0.05 for EQ-5D, −9.27 for EQ-VAS and −0.13 for SF-6D) after controlling for socio-economic characteristics. SAQ scores showed that stable angina patients experienced impaired disease-specific health status, especially in angina stability (40.5±34.6). Nested regressions indicated stable angina-specific health status explained most of the variation in HRQoL, among which disease perception, physical limitation, and angina stability were the strongest predictors. More physical exercise and better sleep were positively related with HRQoL.

**Conclusions:**

Compared to the general population, stable angina patients were associated with lower HRQoL and lower health utility scores, which were largely impacted by clinical symptoms. Further studies are needed to characterize the influence of geographic and cultural factors on the variations of health-related utility in stable angina patients.

## Introduction

Stable angina, the cardinal symptom of coronary artery disease (CAD), is prevalent with an annual incidence of about 0.5% in western populations ≥ 40 years old [1]. It is characterized by a growth in incidence with age and a slightly higher prevalence in women [2], [3]. Stable angina is estimated to affect 30–40 thousand per million people in Europe and the US [1], [4]. In China, approximately 7.7 thousand per million people have CAD [5] and about half of them suffer from angina [6], [7]. Among patients with stable angina who are seen in primary care, 29% experience one or more episodes of angina per week, which indicates diminished quality of life, loss of productivity, and disability [8].

Stable angina is a major debilitating health condition with common chronic symptoms of intermittent, reversible chest pain or discomfort. It has a major negative impact on health-related quality of life (HRQoL), including poor general health status, pain, impaired role functioning, activity restriction, inability to self-manage, and psychological distress [9]–[11]. As such, one of the main treatment goals of stable angina is to achieve optimal symptom control and to improve HRQoL. The most commonly used drug categories to relieve angina symptoms are nitrates, beta blockers, and calcium channel blockers, but continuous use of nitrates in the long-term induces tolerance, with diminishing therapeutic effect [12]. Therefore, it would be important to measure patient health status and to monitor disease progression and response to treatment. However, few studies measured improvements in quality of life on anti-angina therapy [13], [14].

Clinical studies often include disease-specific outcome measures [15]–[17]. For example, the Seattle Angina Questionnaire (SAQ) is a commonly used disease-specific HRQoL measure for stable angina, in which scores for multiple domains are produced [18]. However, SAQ does not reflect how individuals perceive the impact of angina-symptoms on their HRQoL. For optimal resource allocation, the impact of interventions on HRQoL is ideally measured in generic preference-based tools in order to facilitate comparison across different conditions and patient groups [19]–[21]. However, few HRQoL studies on patients with stable angina used generic preference-based measures [14], [22], especially for Chinese patients.

Since HRQoL impairment is an important adverse outcome of stable angina, measuring HRQoL and exploring the associated factors can assist healthcare providers in making better clinical decisions for treatment. In addition, as the management of stable angina patients could potentially involve substantial resource utilization [15], information on outcome measures is particularly important for assessing the cost-effectiveness of new drugs or approaches in angina care. In this light, we used two preference-based HRQoL instruments, EuroQoL (EQ-5D plus visual analogue scale EQ-VAS) and Short Form 6D (SF-6D) to evaluate HRQoL and health-related utility in Chinese patients with stable angina for this study.

The purpose of this study was to describe HRQoL of Chinese stable angina patients using generic outcome measures and compare that to the general population. We hypothesized that Chinese patients with stable angina would experience impaired HRQoL and lower health utility, compared with the general population. We also hypothesized that various factors such as age, gender, the prevalence of weekly angina symptoms, self-perception of illness, symptom clusters, compliance, and exercise tolerance were potential determinants of HRQoL in Chinese patients with stable angina, as indicated by current literature [23]–[26]. We intended to explore how these factors may influence HRQoL in these patients.

## Methods

### Ethics Statement

The study protocol was approved by the Institutional Review Board (IRB) of Tianjin University, and written informed consent concerning the conduct of the survey was obtained from each subject before participating in the study. Survey participants were de-identified and participation in the study was totally voluntary.

### Study design and patient recruitment

The study recruited patients from two tertiary hospitals in Tianjin and two community health service centers in Chengdu from July through December 2011. The two study cities, Tianjin from northern China and Chengdu from southern China, were chosen as an attempt to obtain a more representative sample. Tianjin is a city of district-wide authority with per capita GDP of about 85,000 Chinese Yuan (equivalent to 13,000 US dollars); Chengdu is a provincial capital with per capita GDP of about 59,000 Chinese Yuan (equivalent to 9,100 US dollars) in 2011 [27]. The community health service centers where stable angina patients in Chengdu were recruited are equivalent to outpatient clinics, which administer chronic disease management programs and are the usual source of care for stable angina patients locally.

Patients were included in the study if they were 18 years or older and had been clinically diagnosed with stable angina by their attending physician based on clinical symptoms and examinations of coronary angiography, dual source Computer Tomography (CT) or history of CAD. Angina diagnosis was defined as the association of chest pain at rest or on exertion with one positive finding from a cardiovascular examination such as arteriography, scintigraphy, exercise testing, or resting electrocardiogram (ECG). Additional criterion included typical angina symptoms with a report of at least one episode of chest pain in the previous 3 months.

Patients were excluded from the study if they had experienced acute myocardial infarction or coronary revascularization such as coronary artery bypass grafting surgery and percutaneous intervention in the previous 6 months. Patients were also excluded if they had any active exacerbation of gastrointestinal (GI) problems such as ulcers or were unable to differentiate between their GI symptoms and angina pain. These exclusion criteria were used to help target patients with chest pain that was cardiac in nature.

Similarly, adult patients (18 years or older) without stable angina were recruited from the examination center of one Tianjin hospital and two community centers in Chengdu. The inclusion criteria included the ability to comprehend the survey questions and no apparent cognitive impairment as assessed by the interviewers.

All participants were interviewed in person by trained recruiters with a standardized questionnaire containing EQ-5D, EQ-VAS, and SF-6D. Validity of these HRQoL instruments in the general or disease-specific Chinese population was previously reported [28]–[30]. In addition, socio-economic and comorbid conditions (hypertension, diabetes mellitus, and hyperlipidemia) were collected from all participants. Specific to stable angina patients, disease duration and symptom severity measured by SAQ were also collected. The questionnaire and implementation procedures were identical for all study sites and patients.

### Instruments

#### EQ-5D/EQ-VAS

EQ-5D is a generic, preference-based instrument for describing and measuring HRQoL with five dimensions: mobility, self-care, usual activities, pain/discomfort, and anxiety/depression. Each dimension has three possible levels of response: no problem, some problems, and severe problems. The EQ-5D descriptive system can theoretically define 243 health states, each assigned with a utility score ranging from −0.59 to 1.00. The utility scoring algorithm adopted in this study was developed using the time trade-off (TTO) technique on a random UK general population sample [31]. EQ-VAS is a 20-cm vertical analogue scale ranging from 100 (best imaginable health state) to 0 (worst imaginable health state) to represent overall health. Respondents classify and rate their health status on the day of the survey. The simplified Chinese version of EQ-5D/EQ-VAS in this study is the official version authorized by the EuroQoL Group.

#### SF-6D

The SF-6D was developed from Short Form 36 (SF-36) with six dimensions including physical functioning, role-limitations, social functioning, pain, mental functioning, and vitality [32]. Each dimension has four to six levels of response, which generate 18000 possible health states. The SF-6D utility scoring algorithm used in this study was derived from a representative sample of the UK general population using the standard gambling (SG) method, ranging from 0.29 to 1.00 [32]. The recall period is 4 weeks. Our study adopted the Hong Kong Chinese version of SF-6D that has been validated in the general population of Hong Kong and Mainland China [29], [30].

#### SAQ

The Seattle Angina Questionnaire (SAQ), a 19-item index, is a commonly used disease-specific instrument for assessing symptoms and their impact on daily life with CAD. SAQ comprises five domains: physical limitation (PL), anginal stability (AS), anginal frequency (AF), treatment satisfaction (TS), and the disease perception (DP) [17]. SAQ is scored by assigning each response an ordinary value, from 1 (the lowest level of functioning) to 10 (the highest level of functioning), and summing across items within each of the 5 dimensional scales. Scale scores are then transformed to a scale of 0 to 100 by subtracting the lowest possible scale score, dividing the remainder by the range of the scale, and multiplying that by 100 [33]. Higher scores on SAQ subscales indicate higher levels of functioning/satisfaction and fewer limitations. Each subscale monitors a unique dimension of CAD, so no summary score is generated. The Chinese version of SAQ has been shown to be a valid, responsive, and reliable instrument and has been used in clinical trials [34], [35].

### Statistical analyses

Descriptive statistics were performed on the patient sample and the distribution of EQ-5D/EQ-VAS and SF-6D scores. The distribution of socio-economic characteristics, disease severity, life style and co-morbid conditions was compared between stable angina patients and general population ≥40 years old using Chi-square tests. Means and standard deviations were reported for continuous variables, and count and percentages were reported for categorical variables. Due to the skewed distributions of EQ-5D/EQ-VAS and SF-6D values, Mann-Whitney U-tests were performed to identify overall differences between stable angina patients and the general population and differences within subgroups. For each of the EQ-5D and SF-6D dimensions, Fisher's exact test or Pearson Chi-squared test was performed where appropriate to test for statistically significant differences between stable angina patients and the general population.

The differences in HRQoL between stable angina patients and the general population were further evaluated by using a multivariate linear regression, adjusting for socio-economic factors such as age, gender, BMI, marriage status, working status, education, insurance, household income, and city. The differences in the HRQoL scores between stable angina patients and the general population were compared using minimally important difference (MID), which aims to define the threshold value beyond which a patient perceives a health outcome to be beneficial or of clinical significance. A difference of 0.033 or more has been reported to be clinically important in both EQ-5D and SF-6D utility scores [36], [37], while a difference of 5 or more in EQ-VAS is considered to be clinically important [38].

A nested multivariate linear regression was performed to explore factors associated with variations in the HRQoL scores of stable angina patients. Each of the EQ-5D, EQ-VAS, and SF-6D values was modeled as a dependent variable. According to our hypothesis, the determinants of HRQoL include clinical condition, socio-economic, and life-style factors, which were added as three blocks of independent variables in the regression analyses. A nested multivariate linear regression was used with the goal to understand the magnitude of contribution on HRQoL by each block of variables, namely, clinical factors, socio-demographic factors, and life style factors. A nested regression can provide information on both the impact of individual factors and group factors on patient HRQoL, whereas the conventional multivariate linear regression only provides information on the impact of individual factors. Clinical conditions variables, including duration of CAD, SAQ scores, presence of acute and chronic medical conditions, were entered as block 1 in the first step. Socio-economic factors (age, gender, BMI, insurance type, working status, education, household income, marriage status, and living pattern) were then added as block 2. Lastly, life style factors (smoke, drinking, exercise, and sleep disorder) were added as block 3.

To better interpret the results, age was categorized into five groups (18–44, 45–60, 61–74, and ≥ 75 years), and the severity of stable angina symptom for each dimension was classified as moderate (<50) or severe (≥ 50). An alpha level of 0.05 (based on a two-tailed test) was used to determine statistical significance. All data were managed by EpiData (version 3.1, Epidata Association, Odense, Denmark) and analyzed using STATA (version 12.0, STATA Corp LP, Texas, USA).

## Results

### Patient socio-demographics and HRQoL scales distribution

A total of 417 stable angina patients and 567 patients without stable angina were approached; 411 and 549 were recruited with a response rate of 98.6% and 96.8%, respectively.

The characteristics of participants were presented in Table 1. Compared to the general population ≥ 40 years old, stable angina patients were associated with older age, lower education level, lower household income, and higher rates of acute diseases. Stable angina patients were also more likely to be overweight and more likely to be retired. A higher percentage of stable angina patients had a family history of CAD and suffered from sleep disorders compared to the general population. These differences might affect HRQoL scores and were thus adjusted for when we compared HRQoL scores between groups.

**Table 1:** Characteristics of stable angina patients and the general population [N (%)].

Characteristics	Stable angina patients (n = 411)	General Population (n = 549)	General population ≥40 years old (n = 336)	P^a^
Age (years)				<0.001
≤44	12 (2.9)	286 (52.1)	73 (21.7)	
(45–60)	92 (22.4)	176 (32.1)	176 (52.4)	
(61–74)	170 (41.4)	56 (10.2)	56 (16.7)	
**≥**75	137 (33.3)	31 (5.7)	31 (9.2)	
Gender				0.857
Female	207 (50.4)	274 (49.9)	167 (49.7)	
Male	204 (49.6)	275 (50.1)	169 (50.3)	
BMI(kg/m^2^)				0.010
≤25	255 (62.0)	429 (78.1)	243 (72.3)	
(25–30)	131 (31.9)	104 (18.9)	81 (24.1)	
**≥**30	25 (6.1)	16 (2.9)	12 (3.6)	
Education				0.005
Bachelor and above	41 (10.0)	129 (23.5)	49 (14.6)	
High school	144 (35.0)	260 (47.4)	144 (42.9)	
Middle school	125 (30.4)	87 (15.9)	71 (21.1)	
Primary and below	101 (24.6)	73 (13.3)	72 (21.4)	
Marriage status				<0.001
Single	3 (0.7)	108 (19.7)	3 (0.9)	
Married	320 (77.9)	407 (74.1)	301 (89.6)	
Divorced/Widowed	88 (21.4)	34 (6.2)	32 (9.5)	
Monthly household income (Chinese Yuan)				<0.001
≤2500	102 (24.8)	99 (18.0)	75 (22.3)	
(2500–4500)	183 (44.5)	150 (27.3)	93 (27.7)	
[4500–10000)	109 (26.5)	227 (41.3)	127 (37.8)	
**≥**10000	17 (4.1)	73 (13.3)	41 (12.2)	
Working status				<0.001
Working	50 (12.2)	360 (65.6)	192 (57.1)	
Retired	320 (77.9)	78 (14.2)	77 (22.9)	
unemployed	41 (10.0)	111 (20.2)	67 (19.9)	
Insurance type^b^				<0.001
UEBMI	341 (83.0)	322 (58.7)	206 (61.3)	
URBMI	29 (7.1)	70 (12.8)	42 (12.5)	
NRCMI	36 (8.8)	95 (17.3)	66 (19.6)	
Others	5 (1.2)	62 (11.3)	22 (6.6)	
Living situation				0.028
Living with a Partner	365 (88.8)	475 (86.5)	314 (93.5)	
Alone	46 (11.2)	74 (13.5)	22 (6.6)	
Smoking				<0.001
Non Smoker	265 (64.5)	380 (69.2)	218 (64.9)	
Ex-Smoker	93 (22.6)	28 (5.1)	23 (6.9)	
Current Smoker	53 (12.9)	141 (25.7)	95 (28.3)	
Drinking				<0.001
Non Drinker	304 (74.0)	367 (66.9)	228 (67.9)	
Ex-Drinker	62 (15.1)	26 (4.7)	24 (7.1)	
Current Drinker	45 (11.9)	156 (28.4)	84 (25.0)	
Physical exercise per week				0.772
Yes	297 (72.3)	400 (72.9)	246 (73.2)	
No	114 (27.7)	149 (27.1)	90 (26.8)	
Sleep disorder				<0.001
Yes	143 (34.8)	99 (18.0)	99 (18.0)	
No	268 (65.2)	450 (82.0)	450 (82.0)	
Family history of CAD				<0.001
Yes	136 (33.1)	80 (14.6)	59 (17.6)	
No	275 (66.9)	469 (85.4)	277 (82.4)	
Presence of acute medical condition^c^				<0.001
Yes	64 (15.6)	34 (6.2)	24 (7.1)	
No	347 (84.4)	515 (93.8)	312 (92.9)	

a Chi-square Test between the stable angina patients and general patients age > = 40.

b UEBMI: Urban Employee Basic Medical Insurance; URBMI: Urban Residence Basic Medical Insurance; NRCMI: New Rural Cooperative Medical Insurance; Other insurance type included people with commercial insurance and without insurance.

c Self-reported acute medical conditions included upper respiratory tract infections, vomiting, diarrhea, headache, insomnia, and injuries. Recall periods are 4 weeks.

The EQ-5D, SF-6D and EQ-VAS scores of the general population ≥ 40 years old were higher than that of the stable angina patients for almost all subgroups (not shown in tables). The subgroup of men ≥ 74 years old was the only exception where a statistically significant difference was not detected in the HRQoL scores. The overall utility scores of stable angina patients were lower than that of general population with the mean difference of −0.05 (95% CI: −0.02, −0.08) in EQ-5D, −0.13 (95% CI: −0.11, −0.15) in SF-6D, and −9.27 (95% CI: −7.33, −11.21) in EQ-VAS (Table 2), adjusting for the demographic and socioeconomic characteristics using multivariate linear regressions. We further restricted our sample to patients ≥ 40 years old, where stable angina is more prevalent. Differences in the utility scores of stable angina patients persisted yet were smaller compared to the general population: −0.05 (95% CI: −0.01, −0.08) in EQ-5D, −0.13 (95% CI: −0.11, −0.15) in SF-6D, and −8.99 (95% CI: −6.89, −11.08) in EQ-VAS. All differences were statistically significant based on the Mann-Whitney U-tests (P<0.001).

**Table 2:** Overall scores of HRQoL instruments.

		SAP patient (n = 411)	General population (n = 549)	General population ≥40 years old (n = 336)	Difference(95%CI) (general population vs. SAP patients)	Difference(95%CI) (> = 40 general population vs. SAP patients)
**EQ-5D**	Mean (95% CI)	0.75(0.73,0.77)	0.90(0.88,0.92)	0.86(0.84,0.89)	−0.05(−0.02, −0.08)^a^^,^ b	−0.05(−0.01, −0.08)^a^^,^ b
	Median (IQR^c^)	0.75(0.16)	1.00(0.15)	1.0(0.20)		
**SF-6D**	Mean (95% CI)	0.68(0.67,0.69)	0.85(0.84,0.86)	0.84(0.82,0.85)	−0.13(−0.11, −0.15)^a^^,^ b	−0.13(−0.11, −0.15)^a^^,^ b
	Median (IQR)	0.69(0.16)	0.87(0.11)	0.87(0.14)		
**EQ-VAS**	Mean (95% CI)	71.2(70.0,72.4)	83.9(83.0,84.9)	82.6(81.4,83.8)	−9.27(−7.33, −11.21)^a^^,^ b	−8.99(−6.89, −11.08)^a^^,^ b
	Median (IQR)	70.0(15.0)	85.0(10.0)	85.0(10.0)		

a P<0.001 with Manny-Whitney U-test.

b P<0.001 with multiple linear regression adjusted for age, gender, insurance, work status, income, education, and marriage status.

c IQR: inter-quartile range.

### Distribution of EQ-5D and SF-6D domain responses

The distributions of responses on EQ-5D and SF-6D dimensions were described in Table 3. Compared with the general population ≥40 years old, stable angina patients were more likely to report poorer health on all domains in EQ-5D and SF-6D. More limitations were found in the domains of usual activities, pain/discomfort, and anxiety/depression in EQ-5D and in the domains of physical functions, role limitations, and pain in SF-6D.

**Table 3:** Distribution of EQ-5D and SF-6D results within each domain.

Level	Mobility	Self-care	Usual activities	Pain/discomfort	Anxiety/depression	
EQ-5D (%)						
SAP patients (n = 411)						
1	**346 (84.2)** a	**356 (86.6)**	163 (39.7)	182 (44.3)	181 (44.0)	
2	63 (15.3)	51 (12.4)	**243 (59.1)**	**223 (54.3)**	**219 (53.3)**	
3	2 (0.5)	4 (1.0)	5 (1.2)	6 (1.5)	11 (2.7)	
P	Reference					
General population ≥40 years old (n = 336)						
1	**322 (95.8)**	**324 (96.4)**	**294 (87.5)**	**235 (69.9)**	**250 (74.4)**	
2	11 (3.3)	9 (2.7)	37 (11.0)	89 (26.5)	76 (22.6)	
3	3 (0.9)	3 (0.9)	5 (1.5)	12 (3.6)	10 (3.0)	
P^b^	0.000	0.000	0.000	0.000	0.000	
General population (n = 549)						
1	**535 (97.5)**	**537 (97.8)**	**507 (92.4)**	**431 (78.5)**	**418 (76.1)**	
2	11 (2.0)	9 (1.6)	37 (6.7)	106 (19.3)	120 (21.9)	
3	3 (0.6)	3 (0.6)	5 (0.9)	12 (2.2)	11 (2.0)	
P^b^	0.000	0.000	0.000	0.000	0.000	

Level	Physical function	Role limitation	Social Function	Pain	Mental health	Vitality
SF-6D (%)						
SAP patients(n = 411)						
1	4 (1.0)	109 (26.5)	**120 (29.2)**	37 (9.0)	37 (9.0)	19 (4.6)
2	104 (25.3)	**175 (42.6)**	112 (27.3)	44 (10.7)	**157 (38.2)**	**118 (28.7)**
3	**151 (36.7)**	40 (9.7)	78 (19.0)	97 (23.6)	128 (31.1)	106 (25.8)
4	121 (29.4)	87 (21.2)	68 (16.6)	**129 (31.4)**	66 (16.1)	98 (23.8)
5	25 (6.1)	-	33 (8.0)	95 (23.1)	23 (5.6)	70 (17.0)
6	6 (1.5)	-	-	9 (2.2)	-	-
P		Reference				
General population ≥40 years old (n = 336)						
1	**195 (58.0)**	**260 (77.4)**	**277 (82.4)**	**195 (58.0)**	114 (33.9)	86 (25.1)
2	88 (26.2)	37 (11.0)	27 (8.0)	68 (20.2)	**135 (40.2)**	**155 (46.1)**
3	25 (7.4)	20 (6.0)	14 (4.2)	26 (7.7)	62 (18.5)	53 (15.8)
4	23 (6.9)	19 (5.7)	10 (3.0)	26 (7.7)	17 (5.1)	37 (11.0)
5	3 (0.9)	-	8 (2.4)	14 (4.2)	8 (2.4)	5 (1.5)
6	2 (0.6)	-	-	7 (2.1)	-	-
P^b^	0.000	0.000	0.000	0.000	0.000	0.000
General population (n = 549)						
1	**386 (70.3)**	**446 (81.2)**	**473 (86.2)**	**355 (64.7)**	168 (30.6)	153 (27.9)
2	105 (19.1)	44 (8.0)	40 (7.3)	103 (18.8)	**246 (44.8)**	**253 (46.1)**
3	27 (4.9)	34 (6.2)	18 (3.3)	38 (6.9)	98 (17.9)	88 (16.0)
4	25 (4.6)	25 (4.6)	10 (1.8)	32 (5.8)	28 (5.1)	49 (8.9)
5	4 (0.7)	-	8 (1.5)	14 (2.6)	9 (1.6)	6 (1.1)
6	2 (0.4)	-	-	7 (1.3)	-	-
P^b^	0.000	0.000	0.000	0.000	0.000	0.000

a The bolded numbers indicated the highest percentage among the column.

b Fisher's exact test or Pearson Chi-squared test was performed where appropriate.

### Nested multivariate linear regression analyses

As described earlier, nested multivariate linear regression analyses were conducted on EQ-5D, SF-6D utility scores and EQ-VAS scores as separate outcomes. Three blocks of factors were added to the regressions one by one, including clinical conditions (block 1), socio-demographic (block 2), and life-style factors (block 3). Block 1 variables (clinical factors), which included duration of CAD, SAQ scores, and current acute and chronic medical conditions (hypertension, diabetes mellitus, and hyperlipidemia), explained 22.2% (EQ-5D), 37.2% (SF-6D), and 21.7% (EQ-VAS) of the variation in HRQoL of stable angina patients (Table 4). After adding block 2 (socio-demographic factors), the *R* ^2^ increased significantly for SF-6D utility scores (*P* = 0.016), but not significantly for EQ-5D utility scores and EQ-VAS scores. After block 3 (life-style factors) also added, the *R* ^2^ increased significantly for EQ-VAS scores (*P* = 0.008), but not significantly for EQ-5D utility scores and SF-6D utility scores. The final regression model including all blocks was performed and the results of variables with statistically significant influence only were displayed in Table 5. The effects of clinical symptoms and conditions remained relatively persistent in all models. As expected, patients with longer disease durations and more severe stable angina symptoms (indicated by five SAQ dimensions) reported lower overall HRQoL scores. Differences were found in the domains of physical limitation, angina stability and disease perception. Most of these differences were both clinically important and statistically significant. Chronic medical conditions such as hyperlipidemia also predicted worse HRQoL in EQ-5D utility scores. Lower HRQoL scores were observed among less educated patients, but the difference was not statistically significant. Weekly physical exercise and better sleep were positively correlated with HRQoL among stable angina patients.

**Table 4:** Change in R^2^ for each block of variables in the nested multivariate linear regression analyses (n = 411).

	Change in R^2^		
Blocks	EQ-5D	SF-6D	EQ-VAS
Clinical Factors	**+0.222** ***	**+0.372** ***	**+0.217** ***
Socio-Demographic Factors	+0.040	+0.043*	+0.025
Life Style Factors	+0.021	+0.014	+0.037**

***P<0.001; **P<0.01; *P<0.05.

**Table 5:** Nested multivariate linear regression analyses for HRQoL scores of stable angina patients (n = 411).

Independent variables^a^	EQ-5D	SF-6D	EQ-VAS
	Coefficient	Coefficient	Coefficient
**Block1 (Clinical Factors)**			
SAQ Physical limitation (<50 vs. ≥50)	**−0.126** ***	**−0.084** ***	−0.332
SAQ Angina stability (<50 vs. ≥50)	**−0.065** ***	**−0.035** ***	−2.003
SAQ Angina frequency (<50 vs. ≥50)	−0.007	−0.006	−3.640**
SAQ Treatment satisfaction (<50vs. ≥50)	−0.037	**−0.037** ***	−3.019*
SAQ Disease perception (<50 vs. ≥50)	**−0.079** ***	**−0.096** ***	**−6.703** ***
Hypertension (Yes vs. Non)	0.013	−0.017*	−1.592
Diabetes mellitus (Yes vs. Non)	−0.038*	−0.017	−2.028
Hyperlipidemia (Yes vs. Non)	**−0.055** ***	−0.014	−0.776
**Block2 (Socio-Demographic Factors)**			
Gender (Male vs. female)	0.022	0.022*	−0.309
Insurance type (vs. UEBMI)			
URBMI & NRCMI & Others	−0.004	0.036**	−0.098
Education (vs. Bachelor and above)			
High school	−0.015	−0.023	−0.969
Middle school	−0.016	−0.034**	−0.809
Primary and below	0.004	−0.032*	−3.206
Living pattern			
Alone(vs. Living with a Partner)	−0.089**	−0.012	−2.002
**Block3 (Life Style Factors)**			
Doing exercise per week (No vs. yes)	−0.038*	−0.024**	−2.936**
Sleep disorder (Yes vs. non)	−0.035*	−0.011	−2.108*
**R^2^**	0.283	0.429	0.283

***P<0.01; **P<0.05; *P<0.10.

a Only variables with statistically significant results were displayed. Except variables listed in the table, other explanatory variables included duration of CAD and presence of acute medical condition in block1, age, BMI, working status, household income, and marriage status in block 2, smoke, and drinking for block 3.

## Discussion

In the current study, we used two generic preference-based instruments (EQ-5D including EQ-VAS and SF-6D) and one disease-specific profile (SAQ) to assess HRQoL of Chinese patients with stable angina. We investigated the effects of clinical, socio-economic, and life-style factors on health-related utility scores. Our results suggested that health-related utility scores were significantly lower in patients with stable angina when compared to the general population adjusting for age. Clinical symptoms indicated by SAQ (all five domains) were the main predictors in decreased HRQoL scores of stable angina patients. Our findings provided further evidence of HRQoL impairment among stable angina patients and identified potential new variables that may be modulated to improve HRQoL in these patients, such as treatment satisfaction and physical exercise. To our knowledge, this is also the first paper applying multiple preference-based HRQoL measures (EQ-5D and SF-6D) to measure utility in Chinese patients with stable angina.

In this cross-sectional study, the overall utility scores of stable angina patients were significantly lower than that of the general population. The magnitude of differences exceeded previously reported MID of 5 for EQ-VAS and 0.033 for SF-6D, however, there was insufficient MID (0.074) for EQ-5D [37], [38]. According to existing literature, the health-related utility of the general population is estimated to be 0.85 in EQ-5D and 0.81 in SF-6D in two different cities of China [39] and the EQ-VAS scores are estimated to range from 78.3 to 83.7, possibly due to geographical variations [39]–[41]. Compared with the previous studies, the EQ-5D (0.90), SF-6D (0.85), and EQ-VAS (83.9) scores of the general population in our study appeared to be relatively high. Possible explanations of these discrepancies include differences in study design and study populations. To our best knowledge, the norms of EQ-5D and SF-6D for the general population in China have not been established and further research in this topic is needed.

When compared with the general population of all ages and of those ≥40 years old, the lower EQ-5D and SF-6D ratings of stable angina patients in the domains of physical limitation, pain, and anxiety/depression indicated their impact on patients' HRQoL (Table 3). This is consistent with previous findings that physical limitation, pain, and psychological problems lead to significant HRQoL impairment among stable angina patients [25], [42].

The health-related utility of stable angina patients in our study was estimated at 0.75 in EQ-5D, very similar to the results from Kiessling and Henriksson in Sweden (0.76 in EQ-5D). However, the estimated EQ-VAS score was 0.75, higher than the same study (0.68 in EQ-VAS) [43]. With no reference utility scores of SF-6D from other countries, we were not able to compare the variations in utility scores. However, we cannot rule out the possibility of utility variations due to different geographic and cultural backgrounds.

The use of preference-based measures of health outcomes makes it possible to incorporate HRQoL and length of survival into a single measure, such as quality-adjusted life years (QALY), which is a fundamental component in cost-utility analysis (CUA). CUA has been formally adopted by many countries in health technology evaluation to inform reimbursement decisions.

In the nested regression analyses, with and without adjusting for socio-economic and life style factors, clinical factors were the only major factors associated with lower HRQoL in all three instruments (Table 4&5). The results differed from other studies where age, gender, and angina frequency were significant predictors of HRQoL [22], [25]. However, we did not observe a statistically significant impact of angina frequency on EQ-5D and SF-6D, and only observed limited significance in EQ-VAS (P = 0.058, Table 5). One possible explanation for this finding is that stable angina patients were recruited from outpatient facilities, so their condition may have been less severe. Indeed, we observed different explanatory factors in the SAQ instruments for EQ-VAS scores, compared to SF-6D and EQ-5D scores. However, it may also reflect a true difference among stable angina patients' experience in HRQoL impairment among different countries and cultural backgrounds. The higher R^2^ and more statistically significant explanatory variables in SF-6D utility regression indicated that SF-6D may be a more effective tool to measure HRQoL in angina patients.

Finally, the strengths of this study include (1) the use of multiple preference-based HRQoL measures, EQ-5D, and SF-6D, in Chinese stable angina patients, (2) comparisons with the general population, and (3) exploration of potential predicting factors of HRQoL among patients with stable angina. Our evaluation of HRQoL in stable angina patients confirmed there were impaired dimensions of quality of life and adverse impacts on their well-being in these patients. Furthermore, by identifying potential factors associated with their overall health utility, it may be possible to plan subsequent healthcare interventions to improve HRQoL of the most vulnerable patients.

This study has several limitations. First, as a cross-sectional study, it cannot draw causal inferences between various factors and HRQoL among stable angina patients. Second, utility estimates using the UK standardized algorithm might not have been completely suitable for the Chinese population; however, that was the best estimate available [44]. A third limitation is that the general population in this study were recruited from Physical Examination Centers and were associated with lower age, better education, higher employment rates, and higher income. Therefore it was not a nationally representative sample. Noticeably, even though these two generic HRQoL questionnaires can be used for comparisons between different patient groups and can produce utility indices for economic evaluations, they are not disease specific and some of the detrimental effects observed in the stable angina patients might not be included in the evaluation. Thus, a nation-wide survey with a larger sample size and a careful sampling design is needed to generate the norms of EQ-5D and SF-6D utility index in China, for future research.

## Conclusion

In conclusion, by using two preference-based HRQoL instruments, EQ-5D and SF-6D, we found that stable angina patients experienced reduced HRQoL with lower health utility scores compared to the general population. The scores on physical limitations and pain predicted decreased health-related utility. However, further research is needed to provide reference utility scores of the general population for comparisons and to better characterize the influence of geographic and cultural factors on health-related utility of stable angina patients in China.
